# COVID-19 Vaccine-Associated Lymphadenopathy in Breast Imaging Recipients: A Review of Literature

**DOI:** 10.7759/cureus.26845

**Published:** 2022-07-14

**Authors:** Roxanne T Aleman, Julia Rauch, Janvi Desai, Joumana T Chaiban

**Affiliations:** 1 Internal Medicine, Advocate Christ Medical Center, Oak Lawn, USA; 2 Internal Medicine, Chicago Medical School, Rosalind Franklin University of Medicine and Science, North Chicago, USA; 3 Internal Medicine, University of Illinois College of Medicine, Chicago, USA; 4 Endocrinology, Diabetes and Metabolism, Advocate Christ Medical Center, Oak Lawn, USA

**Keywords:** covid-19 vaccine side effects, covid-19 vaccine-associated lymphadenopthy, lymph nodes, adenopathy, breast cancer detection, reactive lymphadenopathy, screening mammogram, covid-19 vaccine, breast screening, covid-19

## Abstract

The unpredictability of the coronavirus disease 2019 (COVID-19) pandemic has created an ongoing global healthcare crisis. Implementation of a mass vaccination program to accelerate disease control remains in progress. Although injection site soreness, fatigue, and fever are the most common adverse reactions reported after a COVID-19 vaccination, ipsilateral lymph node enlargement has increasingly been observed. In patients undergoing routine screening and surveillance for breast cancer, interpreting lymphadenopathy (LAP) is challenging in the setting of a recent COVID-19 vaccination. With a growing proportion of the population receiving the vaccine, a multifaceted approach is necessary to avoid unnecessary and costly workup. In this comprehensive review, we summarize the existing literature on COVID-19 vaccine-associated LAP in breast imaging patients.

## Introduction and background

Coronavirus disease 2019 (COVID-19) is a highly contagious infection caused by the SARS-CoV-2 virus discovered in Wuhan, China, in December 2019 [1]. On March 11, 2020, the World Health Organization (WHO) declared this rapidly spreading illness a global pandemic [2]. 

The worldwide spread of the virus and its rapid increase in mortality necessitated the expeditious development of a novel vaccine. Early December 2020 marked the beginning of the first mass vaccination program [3]. There are 10 COVID-19 vaccines approved for use by the WHO. These include Oxford-AstraZeneca (AstraZeneca), Johnson and Johnson’s Janssen (J&J), Moderna, Pfizer-BioNTech (Pfizer), Sinopharm, Sinovac, COVAXIN, Covovax, Nuvaxovid, and CanSino [4]. Pfizer, Moderna, and J&J have been approved for emergency use by the United States Food and Drug Administration (FDA). The Centers for Disease Control and Prevention (CDC) is currently recommending the primary vaccine series for those aged six months and older and, if eligible, boosters for those five years and older [5]. 

As of June 17, 2022, the CDC online COVID tracker reported that 78.1% of the United States population had received at least one dose of the COVID-19 vaccine, while 66.8% are considered fully vaccinated. Additionally, 47.2% of those considered fully vaccinated have been administered the first booster dose [5]. While the primary vaccine and boosters are deemed safe and effective, increased reports of adverse events are inevitable with the execution of mass vaccination. 

Clinical and radiologic evidence of transient reactive lymph node enlargement secondary to the COVID-19 vaccinations is well documented in the literature [6-8]. Clinical signs of lymphadenopathy (LAP) following COVID-19 vaccination have been noted to include lymph node swelling and tenderness ipsilateral to the site of injection [9]. Meanwhile, radiologic evidence of LAP following COVID-19 vaccination, observed on various imaging modalities, have been noted to include diffuse and cortical lymph node thickening [10]. The presence of LAP raises the question if this is due to one’s immune system reacting to the vaccine versus an underlying malignant process, infection, autoimmune condition, or medication. This article aims to synthesize the available data on COVID-19 vaccine-associated LAP in breast imaging recipients and to reduce the use of unneeded imaging and invasive procedures in these patients.

## Review

Methods 

Selection Criteria and Search Strategies 

A comprehensive literature search was performed by three authors (RTA, JR, JD) using scientific databases including PubMed, Google Scholar, and Science Direct. Search strings included “COVID-19” AND “vaccine” AND “lymphadenopathy” AND “mammogram” OR "mammography" OR “breast imaging” OR “breast MRI.” No MeSH terms were utilized. The following study designs were included in our final review: retrospective, case series, and case reports. Pre-existing literature reviews and systematic reviews were excluded. All articles were reviewed for relevancy by reading the title and abstract. After removing duplicate articles, we included data from 26 studies relevant to our topic. We included retrospective observational studies, case series, and case reports published in English. Many of these articles included patients with breast imaging such as mammography (MMG), breast ultrasounds (US), magnetic resonance imaging (MRI), and positron emission tomography/computed tomography (PET/CT). The table in Appendices comprises a list of articles used in this report and briefly describes each. 

Data Collection 

Data extraction was completed independently by three authors (RTA, JR, JD). These studies examined characteristics such as age, prior history of breast cancer, malignant findings, and adenopathy location in addition to variables such as imaging type, vaccination type, days since the last COVID-19 vaccination, and whether patients received the first or second dose of the vaccine.

Results 

In our literature review, 26 published (11 retrospective studies, eight case reports, and seven case series) articles were included (Tables 1; table in the Appendices). An analysis of these articles can be seen in Table 1.

**Table 1:** Characteristics and main findings COVID-19: coronavirus disease 2019; MMG: mammography; CT: computed tomography scan; US: ultrasound; MRI: magnetic resonance imaging; PET: positron emission tomography; FDG: fluorodeoxyglucose; NK: not known

Authors	Study design	Imaging type	No. of total patients	No. of patients with adenopathy	Age (mean and range)	No. of previous history of breast cancer	Vaccine type	No. of first dose only	No. of second dose	Adenopathy location	No. of days since last COVID-19 vaccine	No. of new malignant finding
Raj et al., 2022 [11]	Retrospective	MMG	1027	43	Moderna (63.7); Pfizer (59.7); No vaccine (56.4)	NK	Moderna (n=158); Pfizer (n=144); no vaccine (n=725)	NK	NK	Axillary	NK	1
Faermann et al., 2021 [12]	Retrospective	MMG (n=2); US (n=125); MRI (n=36)	163	163	NK	28	Not specified	NK	NK	Ipsilateral axillary	NK	NK
Mehta et al., 2021 [13]	Case series	US (n= 1); MMG + US (n=3)	4	4	50 (42-59)	0	Moderna (n=1); Pfizer (n=3)	3	1	Axillary	5-13	0
Dominguez et al., 2021 [14]	Case report	MMG	1	1	38	0	Pfizer	1	-	Axillary	3	0
Locklin and Woodard, 2021 [15]	Case series	MMG (n=3)	3	3	36-83	0	NK (n=3)	NK	NK	Axillary	1-11	0
Chan and Fischer, 2022 [16]	Care report	MMG	1	1	55	0	Pfizer	-	1	Axillary	14	0
Washington et al., 2021 [17]	Case report	MMG	1	1	37	0	Moderna	1	-	Left axillary and intramammary	12	0
Mortazavi, 2021 [18]	Retrospective	MMG (n=5); US (n=12); MMG + US (n=4); MRI (n=2)	23	23	49 (28-70)	NK	Moderna (n=5); Pfizer (n=12); not specified (n=6)	NK	NK	Axillary	2-6 (n=7); 7-13 (n=7); 14-20 (n=5); >20 (n=1)	NK
Robinson et al., 2021 [19]	Retrospective	MMG	750	23	64 (35-83)	1	Moderna (n=446); Pfizer (n=290); NK (n=14)	4	18	Axillary	1-28	0
Wolfson et al., 2022 [20]	Retrospective	MMG US	1217	537	54.6 (22-90)	76	Moderna (n=459); Pfizer (n=505); J&J (n=18); NK (n=235)	NK	NK	Axillary	1-71	4
Özütemiz et al., 2021 [7]	Case series	PET/CT (n=2); MRI (n=1); MMG + US (n=1)	4 (1 case excluded as not relevant);	4	43.2 (32-57)	1	Pfizer (n=3)	1	3	Axillary (n=4); supraclavicular (n=1)	5-8	0
Lane et al., 2021 [21]	Case series	MRI (n=3); PET/CT (n=3)	6	6	57 (44-76)	6	Moderna (n=2); Pfizer (n=3); NK (n=1)	3	3	Axillary	2-15	0
Lim et al., 2021 [22]	Case series	MRI + US (n=1); US (n=3); MMG + US (n=1); unspecified (n=1)	6	6	67.2 (61-75)	6	AstraZeneca (n=5); Pfizer (n=1)	5	1	Axillary	14-28	0
Park et al., 2022 [23]	Retrospective	US	413	202	44 (17-79)	NK	Pfizer (n=330); AstraZeneca (n=64); Moderna (n=19)	98	104	Axillary	1-14 days (n=77); 15-28 days (n=82); 29-42 (n=29); >43 (n=14)	NK
Plaza et al., 2021 [24]	Case report	MMG	1	1	63	0	NK	-	1	Axillary	6	0
Brown et al., 2021 [25]	Case series	FDG PET/CT	4	4	66 (48-83)	4	NK	NK	NK	Axillary	14-21	0
Horvat et al., 2022 [26]	Retrospective cohort	MRI	357	104	Median 51 years	73	Pfizer (n=175); Moderna (n=137); J&J (n=1); NK (n=44)	NK	NK	Axillary	1 dose: 4-30 2 doses: 1-62	3
Duke et al., 2021 [27]	Case series	MMG (n=1); US (n=2); MRI (n=1)	4	4	43.7 (40-50)	1	Moderna (n=1); Pfizer (n=1); NK (n=2)	NK	NK	Axillary	2-23	0
Mori et al., 2022 [28]	Case report	US	1	1	30	0	Pfizer	1	-	Axillary	9	0
Schapiro et al., 2021 [29]	Case report	FDG PET CT	1	1	48	1	Moderna	1	-	Axillary subpectoral	7	0
Woodard and Zamora, 2021 [30]	Case Report	MRI, US	1	1	45	0	NK	0	1	Axillary	1	0
Eifer et al., 2022 [31]	Retrospective	PET/CT	426	178	67 (20-95)	NK	Pfizer	NK	NK	Axillary	1-34	NK
Cohen et al., 2021 [32]	Retrospective	PET/CT	728	332	69.2 (61.1-76.2)	113 (15.5%)	Pfizer	346	382	Axillary and supraclavicular	1st dose: 0-5 (n=54); 6-12 (n=106); 13+ (150); 2nd dose: 0-6 (n= 76); 7-19 (n= 175); 20+ (n= 100)	17
Nguyen et al., 2022 [33]	Retrospective	US	94	94	56.0 (43.6- 68.4)	26	Moderna (n=45); Pfizer (n= 42); J&J (n= 1); NK (n=6)	NK	NK	Axillary	<13 (n= 33); > or = 3 (n=61)	3
Lam and Flanagan, 2022 [34]	Case report	MRI, US	1	1	39	1	Pfizer	0	1	Axillary	1	0
Bernstine et al., 2021 [35]	Retrospective	PET/CT	650	168	68.7 (20-97)	95	Pfizer	394	256	Axillary	1-22 days	NK

Discussion

LAP reports will likely increase as the COVID-19 vaccine reaches a broader patient population. With increasing vaccination rates, side effects from vaccination are expected to become more noticeable, and thus more likely to be reported. The purpose of this literature review was to summarize the available data related to LAP after receiving at least one dose of the COVID-19 vaccine. It is essential to consider time variation, the number of vaccinations received, and personal patient characteristics when LAP is reported on breast imaging. 

LAP Characteristics 

Across the 26 studies reviewed, a total of 5,162 patients received at least one dose of the COVID-19 vaccine, with 1,906 patients (36.92%) showing signs of post-vaccination LAP [7,11-35]. Axillary LAP was seen across all studies, while supraclavicular, intramammary, and subpectoral LAP was also noted, though less frequently [7,17,29,32]. LAP was found through various imaging modalities, including MMG, US, MRI, and PET/CT, with and without fluorodeoxyglucose (FDG) tracing. 

Timing 

Studies that reported the number of days since the last COVID-19 vaccination showed that LAP typically occurs within a month after vaccination. Considering the close timing after vaccine administration, LAP found on breast imaging after COVID-19 vaccination may not merit an aggressive workup. A thorough history and last vaccination date should therefore be taken before an aggressive workup is initiated. A retrospective case series by Robinson et al. found that patients who had received a COVID-19 vaccination within 90 days had a higher incidence of axillary adenopathy present on MMG [19]. The study identified 23 out of 750 cases of axillary adenopathy (3%), much higher than the 0.02-0.04% rate of adenopathy reported in normal MMG, particularly in the first two weeks following vaccination. Additionally, no instances of axillary adenopathy were identified in those who were observed 28 days post-vaccination [19]. 

Vaccine Type 

While vaccinations against HIN1 Influenza, tuberculosis (TB), smallpox, measles, and human papillomavirus (HPV) are associated with regional LAP to varying degrees, post-vaccination LAP is an infrequent adverse effect in the aforementioned vaccinations [9,36-38]. Meanwhile, this effect has been observed with higher frequency in SARS-CoV-2 mRNA vaccine recipients [39]. The two mRNA COVID-19 vaccines, Pfizer and Moderna, were the first mRNA vaccines to be granted authorization by the FDA. Most vaccinations work by using a killed or weakened version of a pathogen to trigger the immune system to recognize and respond to it in the future. Messenger RNA (mRNA) vaccines work differently by using genetically engineered mRNA instead of part of an actual bacteria or virus. When mRNA is introduced into the body, it is displayed on antigen-presenting cells and then travels to regional axillary lymph nodes and initiates a large T- and B-cell response for the development of cellular and humoral immunity. As a result, the mRNA vaccination, unlike previous protein-based vaccinations, elicits a more robust immune response within lymph node germinal centers during antigen presentation [10]. The mRNA vaccinations, Moderna and Pfizer, were the two most frequently administered in the studies included in our review. Studies in which patients were administered AstraZeneca, a viral vector vaccine, and J&J, an adenovector vaccine, were less frequently mentioned. 

Patient Characteristics 

In this literature review, it appears that the women with adenopathy were predominantly between 30 years and 60 years of age. According to the United States Preventive Services Task Force (USPSTF), it is recommended for women 50-74 years old to get MMG every two years [40]. Do clinical professionals have an obligation to pursue aggressive workups if women receive MMG that reveals LAP in the setting of recent vaccination? Before the pandemic, women with LAP on breast imaging were recommended for further evaluation. However, vaccine-associated LAP should be considered to avoid unnecessary workup in this patient population. 

Conservative Approach 

In our literature review, 21 studies investigated whether patients with LAP following COVID-19 vaccination showed evidence of new malignant findings. As a whole, new malignancy findings were rarely reported. These 21 studies identified 1,172 patients with LAP, 28 of whom (2.4%) showed new malignancies on imaging. More specifically, in Horvat et al., among 104 patients with LAP and COVID-19 vaccinations, only three were newly diagnosed with breast cancer [26]. In the study by Cohen et al., 17 out of 332 women had a new breast cancer diagnosis [32]. A majority of the patients undergoing aggressive workup (e.g., biopsy) in these studies did not have evidence of malignancy. Follow-up US is less invasive than other imaging modalities and also did not reveal evidence of malignancy in most cases. Despite being less invasive, ultrasound is, however, less sensitive than biopsy for diagnosing malignancy. Therefore, it is important to acknowledge that false negatives can occur.

Management and Recommendations 

In response to the original guidelines suggested by the Society of Breast Imaging, a large, multidisciplinary team of experts at three of the leading tertiary cancer centers in the United States have come forward with recommendations regarding radiographic imaging and post-vaccination imaging LAP. Their recommendations included the following: whenever possible, cancer-related imaging and screening should be performed before vaccination. As mortality rates due to infection are more significant than the reduction in mortality rates seen from screening, they suggested that patients being screened for cancer who are at increased risk or patients with a known history of cancer should not delay vaccination due to scheduled imaging, as these patients are at higher risk for serious COVID-19 infection and complications. In line with the recommendations by the Society of Breast Imaging in 2021, they suggested that screening MMG should either be scheduled before a patient’s first dose or four to six weeks after the second dose of the vaccine. In addition, the team recommended extending this interval to six weeks after the final vaccination dose, stating that it is common for LAP to remain detectable on imaging at four weeks. Imaging should not be delayed in an acute situation [41]. 

If a patient has cancer or has a known history of cancer, all vaccinations should be administered contralateral to the affected side, in the same location on the arm [32]. Whenever new-onset LAP follows vaccination, Becker et al. recommend observation for six weeks before a thorough diagnostic workup and consider US follow-up if there is a history of cancer. A tissue biopsy should be performed only if there is a concern for metastatic nodal cancer, where prompt identification and treatment are required [41].

Since their initial recommendations in the winter of 2021, the Society of Breast Imaging updated its guidelines as of February 2022 for managing and screening individuals with post-vaccination LAP. It is no longer recommended to delay screening MMG for four to six weeks after the COVID-19 vaccination. A Breast Imaging Reporting and Data System (BI-RADS) category 1 was previously assigned to patients with unilateral axillary LAP on screening MMG with a recent history of COVID-19 vaccination. The latest guidelines recommend categorizing these patients as BI-RADS category 2 (benign), requiring further routine screening. If given a BI-RADS category 3 (probably benign), previous recommendations suggested a follow-up interval of four to twelve weeks. As post-vaccine LAP may persist for a prolonged period, the guidelines now suggest a follow-up interval of longer than twelve weeks. Patients with persistent axillary LAP were previously considered for biopsy. According to the Society of Breast Imaging, patients with improved axillary LAP should be assigned a BI-RADS category 2, or if the condition remains unchanged, a BI-RADS category 3, which will warrant continued follow-up at six months. A lymph node biopsy should only be considered if adenopathy increases [42]. 

It is essential to consider tissue sampling and prompt diagnostic evaluation in patients with LAP and associated breast parenchymal abnormalities. This refined approach may prevent delays in diagnosis and treatment for patients with malignancy masked by symptoms from vaccination. A review by Hao et al. highlights an instance in which a patient with ipsilateral LAP and associated breast parenchymal change (breast edema) seen on MMG twelve days post-vaccination was found to have a metastatic invasive lobular carcinoma on biopsy [43]. Hence, clinical judgment and consideration of associated symptoms are essential when determining whether to perform breast imaging. 

Limitations 

The study's design must be viewed in light of some limitations. A significant limitation is the insufficient sample size for a meaningful statistical analysis. Most of the literature available are case reports and case series. Therefore, we recognize that their findings lack generalization. Furthermore, the minimal cohort studies we found target different variables. This manuscript places all the available literature to date in one article for easy readability. Considerations for future studies with potential for generalizability may include prospective observational studies following patients with post-COVID-19 vaccine LAP over time. 

## Conclusions

Vaccination guidelines and recommendations are constantly evolving as a result of the unpredictability of the new SARS-CoV-2 variants. In the course of promoting booster doses among eligible populations, LAP is expected to increase in frequency. Having reviewed 26 published articles, we are able to appreciate how the presence of LAP after the COVID-19 vaccination can impact clinical decision-making. Maintaining an updated vaccine record and educating patients about less common adverse effects of the COVID-19 vaccine may help to prevent unnecessary imaging and testing for reactive LAP. A further investigation of the incidence of LAP in women after receiving the third dose of the COVID-19 vaccine along with any subsequent changes in mammogram guidelines needs to be explored.

## Appendices

**Table 2:** Published studies used in our literature review COVID-19: coronavirus disease 2019; MMG: mammography; LAP: lymphadenopathy; ED: emergency department; CT: computed tomography scan; C/A/P: chest/abdomen/pelvis; US: ultrasound; MRI: magnetic resonance imaging; BI-RADS: Breast Imaging Reporting and Data System; PET: positron emission tomography; mRNA: messenger ribonucleic acid; FDG: fluorodeoxyglucose; DCIS: ductal carcinoma in situ

S. no.	Authors	Study name	Brief description
1	Raj et al. [11]	COVID-19 vaccine associated subclinical axillary lymphadenopathy on screening mammogram	Retrospective study on 1027 women who underwent screening mammography (MMG) from December 14, 2020, to April 14, 2021. Subclinical axillary lymphadenopathy (LAP) was observed in 13.2% of women who received the Pfizer-BioNTech vaccine versus 9.5% of those who received the Moderna vaccine. Only 1.2% who did not get any Coronavirus 2019 (COVID-19) vaccine showed subclinical unilateral axillary LAP.
2	Faermann et al. [12]	COVID-19 vaccination induced lymphadenopathy in a specialized breast imaging clinic in Israel: analysis of 163 cases	Retrospective observational study of all women who underwent MMG at their breast imaging center from January 11, 2021, to February 4, 2021. Vaccination-induced axillary LAP was seen in 163 women. The study concluded that the number of detected LAPs increased by 394% (p=0.00001) compared to the prior two years.
3	Mehta et al. [13]	Unilateral axillary adenopathy in the setting of COVID-19 vaccine: follow-up	Presents the first four reported cases of patients found to have vaccine-induced unilateral axillary adenopathy as seen on MMG after receiving one or two doses of the Pfizer-BioNTech or Moderna COVID-19 vaccine.
4	Dominguez et al. [14]	Unilateral axillary lymphadenopathy following COVID-19 vaccination: a case report and imaging findings	Case report on a 38-year-old woman presenting to the emergency department (ED) with abdominal pain and 20-pound unintentional weight loss. Received the first dose of Pfizer BioNTech vaccine three days before ED. Computed tomography (CT) chest/abdomen/pelvis (C/A/P) revealed unilateral axillary LAP ipsilateral to vaccine site. The subsequent diagnostic MMG showed no evidence of malignancy and improvement in LAP compared to CT. They did no further workup.
5	Locklin and Woodard [15]	Mammographic and sonographic findings in the breast and axillary tail following a COVID-19 vaccine	Case series on three patients found to have vaccine-associated axillary LAP as seen on MMG and ultrasound (US). Dose number and vaccine type remain unknown. MMG findings such as trabecular and skin thickening, along with increased echogenicity on the US, can be seen with edema secondary to capillary leak or poor lymphatic drainage and should be considered as a possible etiology for the observed breast edema following a recent COVID-19 vaccine.
6	Chan and Fischer [16]	The paralabral cyst: a mimicker of axillary lymphadenopathy in the setting of COVID-19 vaccination	Case report on 55-year-old woman found to have left axillary LAP on MMG two weeks following the second dose of Pfizer COVID-19 vaccine in left deltoid. Follow-up US confirmed reactive axillary lymph node and separate round mass inferomedial to the humeral head. Subsequent shoulder magnetic resonance imaging (MRI) showed inferior lobulated paralabral cyst.
7	Washington et al. [17]	Adenopathy following COVID-19 vaccination	Case report on 37-year-old woman presenting with new-onset palpable left supraclavicular LAP seen after receiving the first dose of Moderna vaccine 12 days prior. The diagnostic MMG showed prominent left axillary and intramammary LAP. A conservative approach using short-term follow-up US was done rather than biopsy.
8	Mortazavi [18]	COVID-19 vaccination-associated axillary adenopathy: imaging findings and follow-up recommendations in 23 women	Retrospective study on 23 women with axillary adenopathy ipsilateral to the vaccinated arms noted on screening or diagnostic breast imaging. In 43% of these women, the adenopathy was discovered incidentally during screening breast imaging (MMG, 5; US, 2; both MMG and US, 1; high-risk screening MRI, 2), and in 43 percent, it was discovered during diagnostic imaging for other reasons (Breast Imaging Reporting and Data System (BI-RADS) category 3 follow-up for breast finding, 3; screening callback for different reason, 2; non-axillary breast pain or lump, 5).
9	Robinson et al. [19]	Incidence of axillary adenopathy in breast imaging after COVID-19 vaccination	Retrospective analysis of 750 women who received one or more COVID-19 vaccinations less than 90 days before getting either a screening or diagnostic MMG between January 15, 2021, and March 22, 2021, at the Jacoby Center for Breast Health in Florida. Twenty-three women (3%) had axillary adenopathy as seen on MMG. Of the 17 US performed at the time of the article, radiology recommendations included no follow-up (n=2), repeat US in three months (n=14), and biopsy (n=1). Biopsy was negative for malignancy.
10	Wolfson et al. [20]	Axillary adenopathy after COVID-19 vaccine: no reason to delay screening mammogram	Retrospective study on 1217 women who received the COVID-19 vaccine and had breast imaging between December 30, 2020, and April 12, 2021. Forty-four percent of the women had LAP identified: 29% on MMG, 61% on US, and 30% on both exams. A biopsy was performed on 8% 43/537 patients. Thirty-four women had benign results, and 9 had concern for malignancy. Four patients were diagnosed with metastatic breast cancer.
11	Özütemiz et al. [7]	Lymphadenopathy in COVID-19 vaccine recipients: a diagnostic dilemma in oncologic patients	Retrospective case series on five cases with ipsilateral axillary LAP occurring after Pfizer-BioNTech from December 21, 2020, to January 27, 2021. Two cases had pathologic confirmation of benign reactive LAP attributed to the vaccination. The remaining three cases were not confirmed histologically but were attributed to recent vaccination administration.
12	Lane et al. [21]	COVID-19 vaccine-related axillary and cervical lymphadenopathy in patients with current or prior breast cancer and other malignancies: cross-sectional imaging findings on MRI, CT, and PET-CT	Case series on six patients who received the COVID-19 vaccination with current or prior malignancy history with adenopathy seen on breast MRI, CT, or positron emission tomography (PET)-CT. They managed two patients with a conservative approach as adenopathy was presumed reactive to a recent COVID-19 vaccination. Two patients had an US-guided biopsy, with both showing benign findings.
13	Lim et al. [22]	COVID-19 vaccine-related axillary lymphadenopathy in breast cancer patients: case series with a review of literature	Case series on six patients with a known history of breast cancer presenting COVID-19 vaccine-related LAP. Demonstrates that interval between COVID-19 vaccination and US detection of LAP ranged from 14 to 28 days (mean of 21.67 days).
14	Park et al. [23]	Axillary lymphadenopathy on ultrasound after COVID-19 vaccinations and its influencing factors: a single-center study	Retrospective study on 413 patients receiving COVID-19 vaccine within twelve weeks prior to US. Axillary LAP was seen in 202 (49%) of these patients. The most important factors included messenger ribonucleic acid (mRNA) type, an interval of four weeks, younger age, and receiving the first dose.
15	Plaza et al. [24]	COVID-19 vaccine-related axillary lymphadenopathy: pattern on screening breast MRI allowing for a benign assessment	Case report on a 63-year-old woman undergoing routine breast screening MRI with axillary LAP seen after a COVID-19 vaccination six days prior. No further workup was done as deemed reactive secondary to the vaccine.
16	Brown et al. [24]	The challenge of staging breast cancer with PET/CT in the era of COVID vaccination	Case series on four breast cancer patients with reactive axillary lymph nodes on fluorodeoxyglucose (FDG) PET/CT. Two patients underwent US-guided biopsy of the lymph node with benign findings. They took a conservative approach on one patient with a follow-up US performed four weeks later.
17	Horvat et al. [26]	Frequency and outcomes of MRI-detected axillary adenopathy following COVID-19 vaccination	Retrospective cohort study on 357 patients receiving COVID-19 vaccine and underwent breast MRI from January 22, 2021, to March 21, 2021. Twenty-nine percent of patients had adenopathy on breast MRI. The most important factors were younger patients and shorter time intervals from receiving the second dose of the vaccine.
18	Duke et al. [27]	Axillary adenopathy following COVID-19 vaccination: a single-institution case series	Case series on four patients with axillary adenopathy on routine screening breast imaging in the setting of recent COVID-19 vaccination (Moderna and Pfizer-BioNTech). Cases show unilateral axillary adenopathy, as well as adenopathy persisting for two to three weeks following vaccination.
19	Mori et al. [28]	Deep axillary lymphadenopathy after coronavirus disease 2019 vaccination: a case report	Case report of a 30-year-old Japanese woman with a case of axillary LAP that occurred nine days after COVID-19 vaccination and mimicked metastasis. She presented with painful axillary masses and axillary LAP was found on US. In follow-up US 14 days after the vaccination, lymph nodes shrank. They noted LAP to be reactive secondary to COVID-19 vaccination.
20	Schapiro et al. [29]	Case report of lymph node activation mimicking cancer progression: a false positive F (18) FDG PET CT after COVID-19 vaccination	This case study shows a false positive F18 FDG PET CT in the left axilla of a woman being treated for metastatic breast cancer after the COVID-19 vaccination. A follow-up US of the axilla indicated no metastasis, indicating that the LAP was likely due to an immune response following vaccination. This case report, in conjunction with prior studies of other vaccines with similar findings, suggests that providers should be aware of potential false-positive imaging following COVID-19 vaccination.
21	Woodard and Zamora [30]	Axillary edema one day after COVID-19 vaccination	Case report on a 45-year-old woman at elevated lifetime risk of developing breast cancer due to strong family history presented for screening breast MRI approximately 24 hours after receiving the second dose of COVID-19 vaccine in the right arm. MRI showed right ipsilateral breast edema and axillary lymph node slightly larger than the contralateral node. US four days post-vaccination showed mild residual edema suggesting initially observed edema on MRI might represent a more acute process related to vaccination.
22	Eifer et al. [31]	Covid-19 mRNA vaccination: age and immune status and its association with axillary lymph node PET/CT uptake.	Retrospective case series of 426 patients receiving the COVID-19 vaccine underwent PET/CT imaging with ipsilateral axillary lymph node uptake seen in 45% of patients on 18F-FDG PET/CT. The number of days from the last vaccine and doses was also significantly associated with increased odds of lymph node uptake. These results were more common amongst immunocompetent patients.
23	Cohen et al. [32]	Hypermetabolic lymphadenopathy following administration of BNT162b2 mRNA Covid-19 vaccine: incidence assessed by [18F] FDG PET-CT and relevance to study interpretation.	Retrospective study of 728 patients receiving Pfizer BNT162b2 mRNA vaccine and underwent [18F] FDG PET-CT studies. The incidence of hypermetabolic lymphadenopathy was 45.6% in patients regardless of their dose. They reported vaccine-associated LAP in 80.1% of patients with HLN. Malignant hypermetabolic axillary or supraclavicular lymph nodes ipsilateral to the vaccination site were interpreted in 5.1% of the vaccinated patients. 14.8 % (49/332) of patients showed equivocal hypermetabolic LAP, with 20 patients in this group being women with breast cancer ipsilateral to the vaccination arm (eight patients at staging).
24	Nguyen et al. [33]	COVID-19 vaccine-related axillary adenopathy on breast imaging: follow-up recommendations and histopathologic findings	Retrospective study describes 94 patients who presented with suspected COVID-19 vaccine-related axillary adenopathy on breast imaging. All biopsies recommended within 12 weeks of the second vaccine dose were benign. In women not recommended for biopsy, the median interval between the second vaccine dose and US follow-up was 15.9 weeks. Three biopsies yielding malignant diagnoses were recommended 12.0-13.1 weeks after the second dose. Lengthening imaging follow-up to 12-16 weeks after the second dose may reduce unnecessary biopsy recommendations.
25	Lam and Flanagan [34]	Axillary lymphadenopathy after COVID-19 vaccination in a woman with breast cancer.	Case report of a 39-year-old woman with known right breast malignancy who underwent MRI before lumpectomy, showing right axillary LAP does not present on prior imaging. The patient reported receiving her second dose of the COVID-19 vaccine in the right arm a day before her breast MRI. Follow-up axillary US performed eight days following MRI showed resolution of LAP. The patient underwent a lumpectomy, and a pathological examination of the excised tissue showed ductal carcinoma in situ (DCIS) with a single focus of microinvasion. Given upgrade to invasive disease, a sentinel lymph node biopsy was performed for staging, and two sentinel nodes were negative, consistent with the diagnosis of vaccination-associated reactive LAP.
26	Bernstine et al. [35]	Axillary lymph nodes hypermetabolism after BNT162b2 mRNA COVID-19 vaccination in cancer patients undergoing 18F-FDG PET/CT	Retrospective cohort study of 651 patients with FDG PET/CT scan for staging or follow-up of cancer following recent COVID-19 vaccinations. Scans found hypermetabolic axillary lymph nodes in 25.8% of scans, divided into two groups: 57 (14.5%) of 394 patients after dose one and 111 (43.3%) of 256 patients after dose two. Therefore, this occurrence was more common after the second injection. LAP was attributed to vaccine injection.
